# Supplementary material for: Epitope editing enables targeted immunotherapy of acute myeloid leukaemia
Source: Nature. 2023 Aug 30;621(7978):404–14. doi: 10.1038/s41586-023-06496-5 (PMC10499609; doi:10.1038/s41586-023-06496-5)
Supplement: Supplementary file 1 — A guide to Supplementary Figs. 1–4 and Supplementary Tables 1–24. [file 41586_2023_6496_MOESM1_ESM.pdf]

nature portfolio

https://doi.org/10.1038/s41586-023-06496-5

Supplementary information

Epitope editing enables targeted immunotherapy of acute myeloid leukaemia

In the format provided by the authors and unedited

Supplementary Information Guide

Supplementary Figure 1 – **A.** Flow cytometry fluorescence minus-one (FMO) controls for FIG.1b and 1g. From left to right, controls for FLT3 staining (Fig.1b left), KIT staining (Fig.1b right) and CD123 staining (Fig.1g) **B**. Uncropped pictures of western blots in FIG.1c. The samples and stimulation conditions are identified with the labels.

Supplementary Figure 2 – **A**. Uncropped pictures of western blots in Extended Data FIG.2a. From left to right, WT FLT3, FLT3 N399D and FLT3 N399G stimulated with different concentrations of FLT3L. **B**. Uncropped pictures of western blots in Extended Data FIG.2b. From left to right, WT KIT and KIT H378R, stimulated with different concentrations of SCF. **C**. Gating strategy for sorting of edited CD34+ HSPCs progenitor fractions in FIG.3b: CD34+133+45RA+90-, CD34+133+45RA-90-, CD34+133+45RA-90+49f-, CD34+133+45RA-90+49f^{mid}.

Supplementary Figure 3 – **A**. List of the NGS sequenced predicted off-target sites for FLT3-sgRNA-18, CD123-sgRNA-R, KIT-sgRNA-Y and FLT3-sgRNA-16. OT sites were identified by searching CRISPOR online tool with each sgRNA sequence, PAM = NRN, Reference genome hg38 and filtered by highest MIT off-target score. Top hits for intronic and exonic regions were selected for targeted deep sequencing. locusDesc indicates the gene name and intronic or exonic location; mismatchPos shows the position of each mismatched base within the sgRNA sequence (*).

Supplementary Figure 4 - **A**. Representative flow cytometry gating strategy for bone marrow analysis of mice xeno-transplanted with edited HSPCs and/or AML PDX (experiment from FIG.5). Cells are pre-gated on singlets, live and physical parameter gates. Populations: 1. human CD45+; 2.T cells (CAR); 3. mNeonGreen+ (AML PDX); 4. human CD45+ w/o CAR or AML; 5. total CD19+; 6. total myeloid cells (CD33/66b+); 7. granulocytes + mast cells; 8. Monocytes; 9. mast cells; 10. total granulocytes; 11. mature granulocytes (CD10+/11c+); 12. immature granulocytes (CD10-/11c-); 13. classical dendritic cells, cDC; 14. pro-B cells; 15. pre-B cells; 16. mature B cells; 17. natural killer cells, NK; 18. Prolymphocytes; 19. Myeloblasts; 20. lin-CD34+ (CD33/66+ lin-CD34+ and CD33/66- lin-CD34+ are pooled for downstream gating); 21. Lin-CD34+38+; 22. Lin-CD34+38-; 23. Pre- B/NK; 24. granulo-mono progenitors, GMP; 25. common myeloid progenitors, CMP; 26. meka-erythroid progenitors, MEP; 27. hematopoietic stem cells, HSC; 28. multipotent progenitors, MPP; 29. lymphoid-primed multipotent progenitors, LMPP; 30. Beads gate; 31. murine CD45+; 32. plasmacytoid DC; 33. multipotent lymphoid progenitors MLP. CountBeads are first gated on singlets as FSC-A^{low}PI^{high} and then with two additional fluorescent parameters (plot in the bottom left corner). **B**. Representative flow cytometry gating strategy for T cell panels on the bone marrow and spleen. Cells are pre-gated on singles, live and physical parameter gates. Human CD45+ cells are separated in CD3+ (T cells) and CD3-, within which the residual AML is gated as mNeonGreen+. CAR+ cells are identified by EGFR staining. T cell phenotype is evaluated on gated CD8+ and CD4+ cells by CD45RA and CD62L. **C**. Representative flow cytometry plots that highlight the discrimination of transduced AML PDX-1 cells (CD33+56+) from healthy CD34+ derived hematopoiesis.

Supplementary Data Table 1 - sequences of 1) gRNAs used in the experiments; 2) primers for PCR amplification and editing quantification; 3) Sleeping Beauty plasmids expressing FLT3, KIT, CD123; 4) Receptor variants; 5) Base editors; 6) ssODN template donors.

Supplementary Data Table 2 - 1) the markers used for gating hematopoietic populations in bone marrow samples; 2) the list of monoclonal antibodies used for flow cytometry, manufacturers, catalog numbers and dilutions used.

Supplementary Data Table 3 – RNAseq of CD34+ HSPCs, list of the DEGs FLT3L vs unstimulated

Supplementary Data Table 4 – RNAseq of CD34+ HSPCs, list of the DEGs IL-3 vs unstimulated

Supplementary Data Table 5 – RNAseq of CD34+ HSPCs, list of the DEGs SCF vs unstimulated

Supplementary Data Table 6 – RNAseq of CD34+ HSPCs, list of the DEGs UT vs AAVS1

Supplementary Data Table 7 – RNAseq of CD34+ HSPCs, Gene Set Enrichment Analyses (GSEA)

Supplementary Data Table 8 – RNAseq of CD34+ HSPCs, KEGG pathway enrichment analysis

Supplementary Data Table 9 – RNAseq of CD34+ HSPCs, Reactome pathway enrichment analysis

Supplementary Data Table 10 – RNAseq of CD34+ HSPCs, list of the DEGs FLT3-BE vs AAVS1

Supplementary Data Table 11 – RNAseq of CD34+ HSPCs, list of the DEGs CD123-BE vs AAVS1

Supplementary Data Table 12 – RNAseq of CD34+ HSPCs, list of the DEGs KIT-BE vs AAVS1

Supplementary Data Table 13 – phospho-MS of CD34+ HSPCs, list of the DEGs FLT3L vs unstimulated

Supplementary Data Table 14 – phospho-MS of CD34+ HSPCs, list of the DEGs IL-3 vs unstimulated

Supplementary Data Table 15 – phospho-MS of CD34+ HSPCs, list of the DEGs SCF vs unstimulated

Supplementary Data Table 16 – Guide-SEQ off-target results

Supplementary Data Table 17 – FLT3 sgRNA-18 predicted OT list and NGS results

Supplementary Data Table 18 – CD123 sgRNA-R predicted OT list and NGS results

Supplementary Data Table 19 – KIT sgRNA-Y predicted OT list and NGS results

Supplementary Data Table 20 – FLT3 sgRNA-16 predicted OT list and NGS results

Supplementary Data Table 21 – RNAseq OT deamination, metrics and coverage for analysis of non-gRNA mediated random deamination reported in Extended Data Fig.8c.

Supplementary Data Table 22 – SNTG1 read count in RNAseq samples from Fig.3g, supporting the lack of expression in hematopoietic progenitors.

Supplementary Data Table 23 – in vivo BM absolute counts of hematopoietic subsets.

Supplementary Data Table 24 – Multiple comparison of dual CAR in vitro killing (Fig.6c).
