# Supplementary material for: Epitope editing enables targeted immunotherapy of acute myeloid leukaemia
Source: Nature. 2023 Aug 30;621(7978):404–14. doi: 10.1038/s41586-023-06496-5 (PMC10499609; doi:10.1038/s41586-023-06496-5)
Supplement: Supplementary file 2 — Reporting Summary [file 41586_2023_6496_MOESM2_ESM.pdf]

nature portfolio

Corresponding author(s): Genovese Pietro

Last updated by author(s): Jun 28, 2023

Reporting Summary

Nature Portfolio wishes to improve the reproducibility of the work that we publish. This form provides structure for consistency and transparency in reporting. For further information on Nature Portfolio policies, see our Editorial Policies and the Editorial Policy Checklist.

Statistics

For all statistical analyses, confirm that the following items are present in the figure legend, table legend, main text, or Methods section.

n/a	Confirmed	
☐	☒	The exact sample size (n) for each experimental group/condition, given as a discrete number and unit of measurement
☐	☒	A statement on whether measurements were taken from distinct samples or whether the same sample was measured repeatedly
☐	☒	The statistical test(s) used AND whether they are one- or two-sided<br>*Only common tests should be described solely by name; describe more complex techniques in the Methods section.*
☐	☒	A description of all covariates tested
☐	☒	A description of any assumptions or corrections, such as tests of normality and adjustment for multiple comparisons
☐	☒	A full description of the statistical parameters including central tendency (e.g. means) or other basic estimates (e.g. regression coefficient) AND variation (e.g. standard deviation) or associated estimates of uncertainty (e.g. confidence intervals)
☐	☒	For null hypothesis testing, the test statistic (e.g. F, t, r) with confidence intervals, effect sizes, degrees of freedom and P value noted<br>*Give P values as exact values whenever suitable.*
☒	☐	For Bayesian analysis, information on the choice of priors and Markov chain Monte Carlo settings
☒	☐	For hierarchical and complex designs, identification of the appropriate level for tests and full reporting of outcomes
☐	☒	Estimates of effect sizes (e.g. Cohen's d, Pearson's r), indicating how they were calculated

Our web collection on statistics for biologists contains articles on many of the points above.

Software and code

Policy information about availability of computer code

Data collection	Immunophenotypic analyses were performed on FACS Fortessa, Fortessa X-20 (BD Pharmingen) or using BDFACS Diva software. Cell sorting was performed on a BD FACS Melody or BD FACS Aria Fusion (BD Biosciences) using BD FACS Diva software v6 or BD FACS Chorus.
Data analysis	EditR v1.0.8 (Kluesner M, Nedveck D, Lahr W, Moriarity B. EditR: A method to quantify base editing via Sanger sequencing. The CRISPR Journal. 2018.); drc R package v3.0; ggplot2 R package v3.4; brglm2 R package v0.9; Bioconductor DESeq2 v1.40.2; R Studio v2022.12.0; R version 4.2.2; FCS express v6 (DeNovo Software); GraphPad Prism v9.4; Microsoft Excel 365, v3201. R software v4.3, R Core Team (2021), R Foundation for Statistical Computing, Vienna, Austria. URL https://www.R-project.org/. STEMvision Software Version 2021.05.07.00.

For manuscripts utilizing custom algorithms or software that are central to the research but not yet described in published literature, software must be made available to editors and reviewers. We strongly encourage code deposition in a community repository (e.g. GitHub). See the Nature Portfolio guidelines for submitting code & software for further information.

Data

Policy information about availability of data

All manuscripts must include a data availability statement. This statement should provide the following information, where applicable:

- Accession codes, unique identifiers, or web links for publicly available datasets
- A description of any restrictions on data availability
- For clinical datasets or third party data, please ensure that the statement adheres to our policy

All relevant data are included in the manuscript, including source data for the figures, RNAseq, GuideSEQ, Off-target analyses,. RNAseq datasets have been deposited with links to BioProject accession number PRJNA986596 in the NCBI BioProject database (https://www.ncbi.nlm.nih.gov/bioproject/). Targeted amplicon sequencing datasets of off-target sites are deposited as BioProject accession number PRJNA986845. All mass spectrometry data files are available for download at: ftp://massive.ucsd.edu/MSV000092272. All remaining data, including source data, are available in this Article and its Supplementary Information.

Human research participants

Policy information about studies involving human research participants and Sex and Gender in Research.

Reporting on sex and gender	*Use the terms sex (biological attribute) and gender (shaped by social and cultural circumstances) carefully in order to avoid confusing both terms. Indicate if findings apply to only one sex or gender; describe whether sex and gender were considered in study design whether sex and/or gender was determined based on self-reporting or assigned and methods used. Provide in the source data disaggregated sex and gender data where this information has been collected, and consent has been obtained for sharing of individual-level data; provide overall numbers in this Reporting Summary. Please state if this information has not been collected. Report sex- and gender-based analyses where performed, justify reasons for lack of sex- and gender-based analysis.*
Population characteristics	*Describe the covariate-relevant population characteristics of the human research participants (e.g. age, genotypic information, past and current diagnosis and treatment categories). If you filled out the behavioural & social sciences study design questions and have nothing to add here, write "See above."*
Recruitment	*Describe how participants were recruited. Outline any potential self-selection bias or other biases that may be present and how these are likely to impact results.*
Ethics oversight	*Identify the organization(s) that approved the study protocol.*

Note that full information on the approval of the study protocol must also be provided in the manuscript.

Field-specific reporting

Please select the one below that is the best fit for your research. If you are not sure, read the appropriate sections before making your selection.

☒ Life sciences ☐ Behavioural & social sciences ☐ Ecological, evolutionary & environmental sciences

For a reference copy of the document with all sections, see nature.com/documents/nr-reporting-summary-flat.pdf

Life sciences study design

All studies must disclose on these points even when the disclosure is negative.

Sample size	Sample size for each experiment was determined by the total number of available treated cells, which is constrained by the human source of the material, to be split among each experimental conditions. Whenever possible we aimed to reach at least 5 replicates per group, thus reaching a minimum and sensible operational criterial for carrying out statistics. In some in vivo experiments, such as secondary transplantation, the total number of available cells was more constrained and limited to what could be harvested from the primary recipients.
Data exclusions	For in vivo analyses of multiplex edited HSPCs, one cage of mice (4 mice) from control group was excluded from further analysis because the immunodeficient animals developed evident signs of an opportunistic infection. No other data or sample were excluded from analysis, unless the data point was missing due to technical reasons (sample availability or instrumentation acquisition errors). All these criteria were pre-established.
Replication	Number of biological replicates is specified for each experiment in figure legends. All attempts at replication were successful. Inferential techniques were applied in presence of adequate sample sizes ($n \geq 5$), otherwise only descriptive statistics are reported.
Randomization	Mice were randomly distributed to each experimental group. All in vitro experiments on cell-lines did not require randomization. Choice of healthy donor for experiments with human T cells or CD34+ HSPCs was random.
Blinding	All reported outcomes are based on measurable variables assessed by user-independent methods/instruments (eg. absolute cell counts by

Blinding	automated counting or CountingBeads, cell immunophenotype by flow cytometry, editing outcomes by genomic Sanger or NGS sequencing). The acquisition of these experimental variables is not affected by blinding.

Reporting for specific materials, systems and methods

We require information from authors about some types of materials, experimental systems and methods used in many studies. Here, indicate whether each material, system or method listed is relevant to your study. If you are not sure if a list item applies to your research, read the appropriate section before selecting a response.

Materials & experimental systems

n/a	Involved in the study
☐	☒ Antibodies
☐	☒ Eukaryotic cell lines
☒	☐ Palaeontology and archaeology
☐	☒ Animals and other organisms
☒	☐ Clinical data
☒	☐ Dual use research of concern

Methods

n/a	Involved in the study
☒	☐ ChIP-seq
☐	☒ Flow cytometry
☒	☐ MRI-based neuroimaging

Antibodies

Antibodies used	See attached Supplementary Table 2 for the complete list of antibodies
Validation	Antibodies targeting FLT3 (clones BV10A4, 4G8), KIT (Fab-79D, 104D2, SR-1, Ab55), CD123 (9F5, 6H6, 7G3, S18016E), which are central to this manuscript were validated on human or murine cell lines overexpressing the target gene by Sleeping Beauty transposase. In particular, K562 cells transduced with the cDNAs of WT human FLT3, CD123 and KIT were used to assess specific binding of clones 4G8, BV10A4, 104D2, 79D, Ab55, 6H6, 9F5, 7G3, S18016F to the intended surface antigen. All antibodies used for the analysis of in vivo samples are commercially available and are validated by the manufacturing companies (Biolegend, BD, R&D) on either human or murine peripheral blood mononuclear cells or cell lines transfected with the appropriate target; antibodies were tested on human peripheral blood mononuclear cells or human bone marrow from healthy donors and titrated to identify optimal staining concentrations.

Eukaryotic cell lines

Policy information about cell lines and Sex and Gender in Research

Cell line source(s)	K562, HEK-293T cells were a kind gift from the Biffi lab (Dana Farber Cancer Institute, Boston, US) and originally obtained from ATCC (CCL-243). NIH-3T3 were a kind gift from the Brendel lab (Boston Children's Hospital, Boston, US) and originally obtained from ATCC (CRL-1658). BaF3 were a kind gift from the Armstrong lab (Dana Farber Cancer Institute, Boston, US) and originally obtained from ATCC (HB-283).
Authentication	None of the cell lines used were authenticated (in this work cell lines were used only to express and study transgenes).
Mycoplasma contamination	All cell lines were tested periodically for mycoplasma contamination and found negative.
Commonly misidentified lines (See ICLAC register)	No commonly misidentified cell lines were used.

Animals and other research organisms

Policy information about studies involving animals; ARRIVE guidelines recommended for reporting animal research, and Sex and Gender in Research

Laboratory animals	NOD.Cg-KitW-41J Tyr + Prkdcscid Il2rgtm1Wjl/ThomJ female mice (aka. NBSGW), 6-8 week old at the time of xenotransplantation with CD34+ HSPCs or AML PDX. Mice were housed in sterile individually ventilated cages and fed autoclaved food and water, with standard 12h day/night light cycle.
Wild animals	The study did not involve wild animals.
Reporting on sex	Only female mice were included for in vivo experiments due to their superior ability to support the engraftment of human CD34+ hematopoietic stem and progenitor cells (Notta F, Doulatov S, Dick JE. Engraftment of human hematopoietic stem cells is more efficient in female NOD/SCID/IL-2Rgc-null recipients. Blood. 2010;115(18):3704–3707)
Field-collected samples	The study did not involve samples collected from the field.
Ethics oversight	All animal experiments were performed in accordance to regulations set by the American Association for Laboratory Animal Science

Ethics oversight	and Dana Farber Cancer Institute (Boston, MA) Institutional Animal Care and Use Committee (IACUC). Approved protocol number: DFCI#21-002.

Note that full information on the approval of the study protocol must also be provided in the manuscript.

Flow Cytometry

Plots

Confirm that:

☒ The axis labels state the marker and fluorochrome used (e.g. CD4-FITC).

☒ The axis scales are clearly visible. Include numbers along axes only for bottom left plot of group (a 'group' is an analysis of identical markers).

☒ All plots are contour plots with outliers or pseudocolor plots.

☒ A numerical value for number of cells or percentage (with statistics) is provided.

Methodology

Sample preparation	Cell lines: harvested, washed, incubated with Fc-block reagent and stained at 4C for 15-30 min, then washed. Mouse peripheral blood: collected, lysed with ACK reagent for 10 minutes at room temperature, then washed (repeat 2x lysis and wash); incubated with human and murine Fc-block, stained at room temperature for 15 minutes, then washed. Bone marrow, spleen: collected, washed, lysed with ACK reagent for 5 minutes at room temperature and washed; incubated with human and murine Fc-block, stained on ice for 35 minutes, then washed. BD Brilliant Stain buffer was included when multiple Brilliant Violet or UV dyes were co-stained. Viability staining was performed using Live/Dead yellow, 7-AAD, Propidium Iodide and AnnexinV staining depending on the experiment. All wash steps were performed with PBS + 2% FBS or PBS + 2% FBS + 2mM EDTA.
Instrument	BD Fortessa flow cytometer, BD Fortessa X-20, BD FACS Melody sorter
Software	BD Diva (acquisition, sorting), BD Chorus (sorting), FCS Express v6 (analysis).
Cell population abundance	BM samples were acquired with a target of 2-4 million total events. The median composition of the total human CD45+ engraftment is as follows: 69% CD19+ 27% CD33/66b+, 2.5% monocytes, 0.42% cDC, 13% immature PMN, 6.7% mature PMN, 0.37% mast cells, 8.9% pro-B, 59% pre-B, 0.57% mature B cells, 0.11% NK, 0.42% B-prolymphocytes, 1.4% myeloblasts, 1.1% lin-CD34+, 0.006% MLP, 0.38% preB/NK, 0.37% GMP, 0.13% CMP, 0.04% MEP, 0.08% LMPP, 0.04% MPP, 0.01% HSC. See Supplementary Data FIG.4 for relative abundance and gating of each populations. We also report absolute counts of all hematopoietic populations from in vivo experiments in the Main Figures and Source Data.
Gating strategy	See Methods, Supplementary Data Table 1 and Supplementary Data FIG.6 for Gating strategies. In vivo BM gating: pre-gating singlets (FSC-A/FSC-H)> live (PI-) > cells (FSC-A/SSC-A)<br>total hematopoietic cells hCD45+ or mCD45+<br>human cells hCD45+<br>murine cells mCD45+<br>AML PDX hCD45+mNeonGreen+<br>T cells (CAR) hCD45+CD3+<br>healthy hematopoiesis (AML and T cells excluded) hCD45+CD3-mNeonGreen-<br>myeloid cells hCD45+CD3-mNeonGreen-CD33/66b+19-<br>total granulocytes hCD45+CD3-mNeonGreen-CD33/66b+19-14-SSChigh<br>immature granulocytes hCD45+CD3-mNeonGreen-CD33/66b+19-14-10-SSChigh<br>mature granulocytes hCD45+CD3-mNeonGreen-CD33/66b+19-14-10+SSChigh<br>monocytes hCD45+CD3-mNeonGreen-CD33/66b+19-14+SSClow<br>cDC hCD45+CD3-mNeonGreen-CD33/66b+19-14-11c+SSClow<br>pDC hCD45+CD3-mNeonGreen-CD33/66b+19-14-11c-123high SSClow<br>myeloblasts hCD45+CD3-mNeonGreen-CD33/66b+19-14-11c-123-34-SSClow<br>CD19+ lymphoid cells hCD45+CD3-mNeonGreen-CD19+CD33/66b-<br>pro-B cells hCD45+CD3-mNeonGreen-CD19+CD33/66b-10+34+<br>pre-B cells hCD45+CD3-mNeonGreen-CD19+CD33/66b-10+34-<br>mature B cells hCD45+CD3-mNeonGreen-CD19+CD33/66b-10-34-<br>NK cells hCD45+CD3-mNeonGreen-CD33/66b-19-56+<br>B-prolymphocytes hCD45+CD3-mNeonGreen-CD33/66b-19-56-34-10+<br>T-prolymphocytes hCD45+CD3-mNeonGreen-CD33/66b-19-56-34-7+<br>lineage-CD34+ hCD45+CD3-mNeonGreen-CD33/66b-19-56-34+ or CD33/66b+19-14-11c-34+SSClow<br>lineage-CD34+38+ lineage-CD34+38+<br>lineage-CD34+38- lineage-CD34+38-<br>pre-B/NK lineage-CD34+38+10+<br>GMP lineage-CD34+38+10-45RA+FLT3+<br>CMP lineage-CD34+38+10-45RA-FLT3+<br>MEP lineage-CD34+38+10-45RA-FLT3-<br>MLP lineage-CD34+38-10+45RA+<br>LMPP lineage-CD34+38-10-45RA+90-<br>MPP lineage-CD34+38-10-45RA-90-

HSC lineage-CD34+38-10-45RA-90+

☒ Tick this box to confirm that a figure exemplifying the gating strategy is provided in the Supplementary Information.
