# Supplementary material for: Epitope editing enables targeted immunotherapy of acute myeloid leukaemia
Source: Nature. 2023 Aug 30;621(7978):404–14. doi: 10.1038/s41586-023-06496-5 (PMC10499609; doi:10.1038/s41586-023-06496-5)
Supplement: Supplementary file 3 — Supplementary Figs. 1–4. [file 41586_2023_6496_MOESM3_ESM.pptx]

## Slide 1
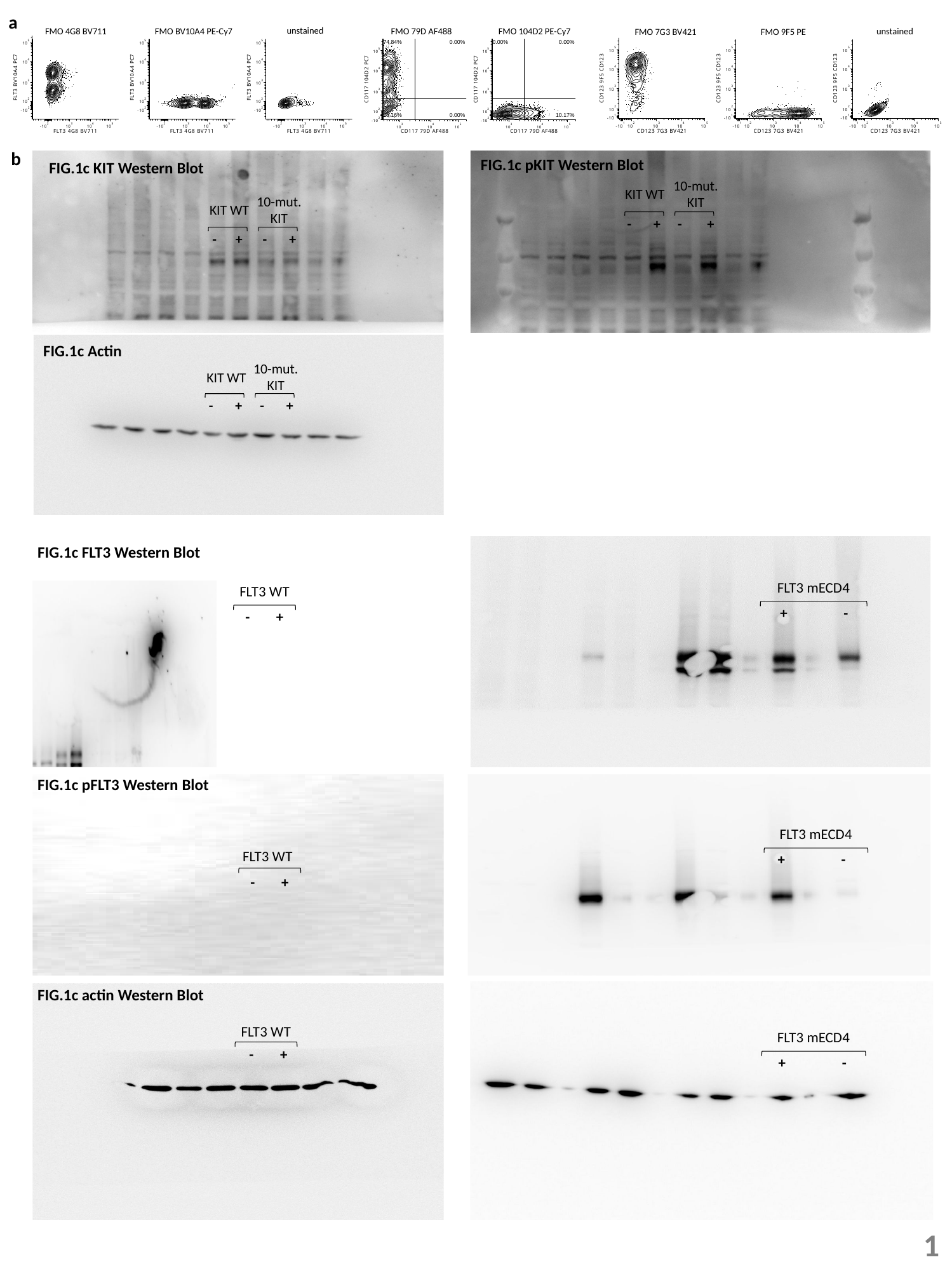

a
unstained
FMO 4G8 BV711
FMO BV10A4 PE-Cy7
FMO 79D AF488
FMO 104D2 PE-Cy7
unstained
FMO 7G3 BV421
FMO 9F5 PE
b
FIG.1c pKIT Western Blot
FIG.1c KIT Western Blot
10-mut. KIT
KIT WT
10-mut. KIT
KIT WT
- +
 - +
- +
 - +
FIG.1c Actin
10-mut. KIT
KIT WT
- +
 - +
FIG.1c FLT3 Western Blot
FLT3 mECD4
FLT3 WT
+ -
- +
FIG.1c pFLT3 Western Blot
FLT3 mECD4
FLT3 WT
+ -
- +
FIG.1c actin Western Blot
FLT3 WT
FLT3 mECD4
- +
+ -
1

## Slide 2
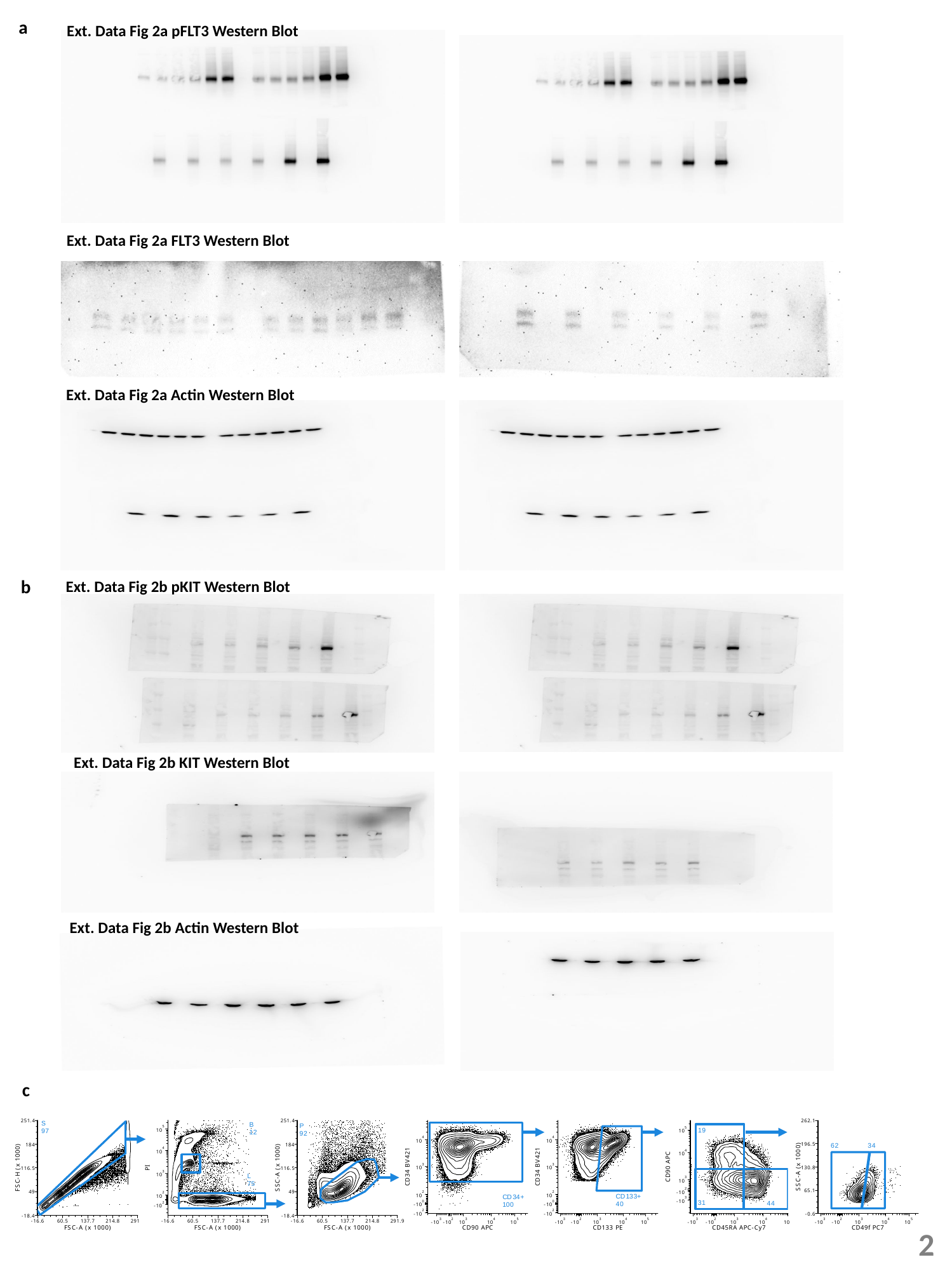

a
Ext. Data Fig 2a pFLT3 Western Blot
Ext. Data Fig 2a FLT3 Western Blot
Ext. Data Fig 2a Actin Western Blot
b
Ext. Data Fig 2b pKIT Western Blot
Ext. Data Fig 2b KIT Western Blot
Ext. Data Fig 2b Actin Western Blot
c
2

## Slide 3
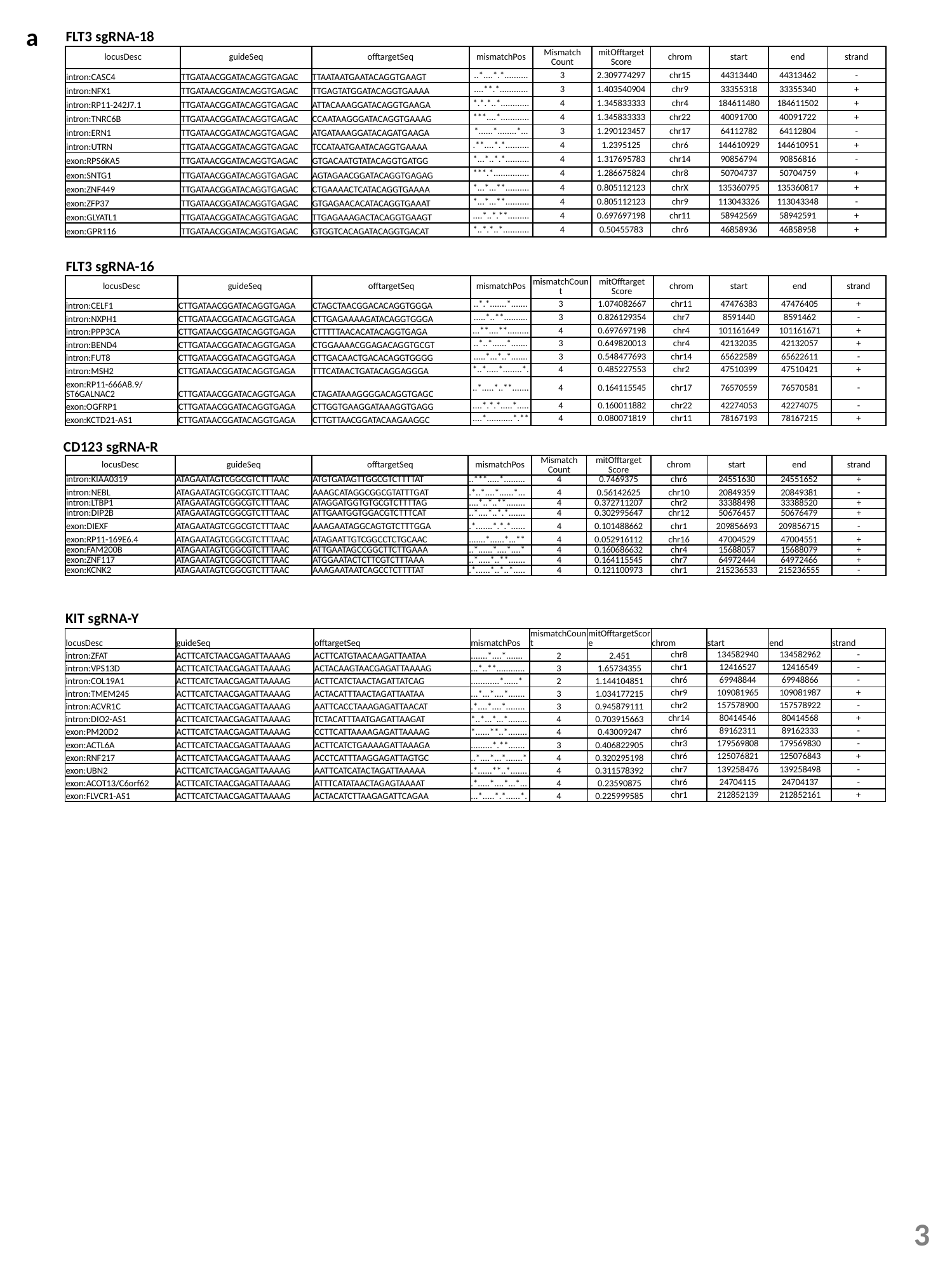

a
 FLT3 sgRNA-18
| locusDesc | guideSeq | offtargetSeq | mismatchPos | Mismatch Count | mitOfftarget Score | chrom | start | end | strand |
| --- | --- | --- | --- | --- | --- | --- | --- | --- | --- |
| intron:CASC4 | TTGATAACGGATACAGGTGAGAC | TTAATAATGAATACAGGTGAAGT | ..\*....\*.\*.......... | 3 | 2.309774297 | chr15 | 44313440 | 44313462 | - |
| intron:NFX1 | TTGATAACGGATACAGGTGAGAC | TTGAGTATGGATACAGGTGAAAA | ....\*\*.\*............ | 3 | 1.403540904 | chr9 | 33355318 | 33355340 | + |
| intron:RP11-242J7.1 | TTGATAACGGATACAGGTGAGAC | ATTACAAAGGATACAGGTGAAGA | \*.\*.\*..\*............ | 4 | 1.345833333 | chr4 | 184611480 | 184611502 | + |
| intron:TNRC6B | TTGATAACGGATACAGGTGAGAC | CCAATAAGGGATACAGGTGAAAG | \*\*\*....\*............ | 4 | 1.345833333 | chr22 | 40091700 | 40091722 | + |
| intron:ERN1 | TTGATAACGGATACAGGTGAGAC | ATGATAAAGGATACAGATGAAGA | \*......\*........\*... | 3 | 1.290123457 | chr17 | 64112782 | 64112804 | - |
| intron:UTRN | TTGATAACGGATACAGGTGAGAC | TCCATAATGAATACAGGTGAAAA | .\*\*....\*.\*.......... | 4 | 1.2395125 | chr6 | 144610929 | 144610951 | + |
| exon:RPS6KA5 | TTGATAACGGATACAGGTGAGAC | GTGACAATGTATACAGGTGATGG | \*...\*..\*.\*.......... | 4 | 1.317695783 | chr14 | 90856794 | 90856816 | - |
| exon:SNTG1 | TTGATAACGGATACAGGTGAGAC | AGTAGAACGGATACAGGTGAGAG | \*\*\*.\*............... | 4 | 1.286675824 | chr8 | 50704737 | 50704759 | + |
| exon:ZNF449 | TTGATAACGGATACAGGTGAGAC | CTGAAAACTCATACAGGTGAAAA | \*...\*...\*\*.......... | 4 | 0.805112123 | chrX | 135360795 | 135360817 | + |
| exon:ZFP37 | TTGATAACGGATACAGGTGAGAC | GTGAGAACACATACAGGTGAAAT | \*...\*...\*\*.......... | 4 | 0.805112123 | chr9 | 113043326 | 113043348 | - |
| exon:GLYATL1 | TTGATAACGGATACAGGTGAGAC | TTGAGAAAGACTACAGGTGAAGT | ....\*..\*.\*\*......... | 4 | 0.697697198 | chr11 | 58942569 | 58942591 | + |
| exon:GPR116 | TTGATAACGGATACAGGTGAGAC | GTGGTCACAGATACAGGTGACAT | \*..\*.\*..\*........... | 4 | 0.50455783 | chr6 | 46858936 | 46858958 | + |
 FLT3 sgRNA-16
| locusDesc | guideSeq | offtargetSeq | mismatchPos | mismatchCount | mitOfftarget Score | chrom | start | end | strand |
| --- | --- | --- | --- | --- | --- | --- | --- | --- | --- |
| intron:CELF1 | CTTGATAACGGATACAGGTGAGA | CTAGCTAACGGACACAGGTGGGA | ..\*.\*.......\*....... | 3 | 1.074082667 | chr11 | 47476383 | 47476405 | + |
| intron:NXPH1 | CTTGATAACGGATACAGGTGAGA | CTTGAGAAAAGATACAGGTGGGA | .....\*..\*\*.......... | 3 | 0.826129354 | chr7 | 8591440 | 8591462 | - |
| intron:PPP3CA | CTTGATAACGGATACAGGTGAGA | CTTTTTAACACATACAGGTGAGA | ...\*\*....\*\*......... | 4 | 0.697697198 | chr4 | 101161649 | 101161671 | + |
| intron:BEND4 | CTTGATAACGGATACAGGTGAGA | CTGGAAAACGGAGACAGGTGCGT | ..\*..\*......\*....... | 3 | 0.649820013 | chr4 | 42132035 | 42132057 | + |
| intron:FUT8 | CTTGATAACGGATACAGGTGAGA | CTTGACAACTGACACAGGTGGGG | .....\*...\*..\*....... | 3 | 0.548477693 | chr14 | 65622589 | 65622611 | - |
| intron:MSH2 | CTTGATAACGGATACAGGTGAGA | TTTCATAACTGATACAGGAGGGA | \*..\*.....\*........\*. | 4 | 0.485227553 | chr2 | 47510399 | 47510421 | + |
| exon:RP11-666A8.9/ST6GALNAC2 | CTTGATAACGGATACAGGTGAGA | CTAGATAAAGGGGACAGGTGAGC | ..\*.....\*..\*\*....... | 4 | 0.164115545 | chr17 | 76570559 | 76570581 | - |
| exon:OGFRP1 | CTTGATAACGGATACAGGTGAGA | CTTGGTGAAGGATAAAGGTGAGG | ....\*.\*.\*.....\*..... | 4 | 0.160011882 | chr22 | 42274053 | 42274075 | - |
| exon:KCTD21-AS1 | CTTGATAACGGATACAGGTGAGA | CTTGTTAACGGATACAAGAAGGC | ....\*...........\*.\*\* | 4 | 0.080071819 | chr11 | 78167193 | 78167215 | + |
 CD123 sgRNA-R
| locusDesc | guideSeq | offtargetSeq | mismatchPos | Mismatch Count | mitOfftarget Score | chrom | start | end | strand |
| --- | --- | --- | --- | --- | --- | --- | --- | --- | --- |
| intron:KIAA0319 | ATAGAATAGTCGGCGTCTTTAAC | ATGTGATAGTTGGCGTCTTTTAT | ..\*\*\*.....\*......... | 4 | 0.7469375 | chr6 | 24551630 | 24551652 | + |
| intron:NEBL | ATAGAATAGTCGGCGTCTTTAAC | AAAGCATAGGCGGCGTATTTGAT | .\*..\*....\*......\*... | 4 | 0.56142625 | chr10 | 20849359 | 20849381 | - |
| intron:LTBP1 | ATAGAATAGTCGGCGTCTTTAAC | ATAGGATGGTGTGCGTCTTTTAG | ....\*..\*..\*\*........ | 4 | 0.372711207 | chr2 | 33388498 | 33388520 | + |
| intron:DIP2B | ATAGAATAGTCGGCGTCTTTAAC | ATTGAATGGTGGACGTCTTTCAT | ..\*....\*..\*.\*....... | 4 | 0.302995647 | chr12 | 50676457 | 50676479 | + |
| exon:DIEXF | ATAGAATAGTCGGCGTCTTTAAC | AAAGAATAGGCAGTGTCTTTGGA | .\*.......\*.\*.\*...... | 4 | 0.101488662 | chr1 | 209856693 | 209856715 | - |
| exon:RP11-169E6.4 | ATAGAATAGTCGGCGTCTTTAAC | ATAGAATTGTCGGCCTCTGCAAC | .......\*......\*...\*\* | 4 | 0.052916112 | chr16 | 47004529 | 47004551 | + |
| exon:FAM200B | ATAGAATAGTCGGCGTCTTTAAC | ATTGAATAGCCGGCTTCTTGAAA | ..\*......\*....\*....\* | 4 | 0.160686632 | chr4 | 15688057 | 15688079 | + |
| exon:ZNF117 | ATAGAATAGTCGGCGTCTTTAAC | ATGGAATACTCTTCGTCTTTAAA | ..\*.....\*..\*\*....... | 4 | 0.164115545 | chr7 | 64972444 | 64972466 | + |
| exon:KCNK2 | ATAGAATAGTCGGCGTCTTTAAC | AAAGAATAATCAGCCTCTTTTAT | .\*......\*..\*..\*..... | 4 | 0.121100973 | chr1 | 215236533 | 215236555 | - |
 KIT sgRNA-Y
| locusDesc | guideSeq | offtargetSeq | mismatchPos | mismatchCount | mitOfftargetScore | chrom | start | end | strand |
| --- | --- | --- | --- | --- | --- | --- | --- | --- | --- |
| intron:ZFAT | ACTTCATCTAACGAGATTAAAAG | ACTTCATGTAACAAGATTAATAA | .......\*....\*....... | 2 | 2.451 | chr8 | 134582940 | 134582962 | - |
| intron:VPS13D | ACTTCATCTAACGAGATTAAAAG | ACTACAAGTAACGAGATTAAAAG | ...\*..\*\*............ | 3 | 1.65734355 | chr1 | 12416527 | 12416549 | - |
| intron:COL19A1 | ACTTCATCTAACGAGATTAAAAG | ACTTCATCTAACTAGATTATCAG | ............\*......\* | 2 | 1.144104851 | chr6 | 69948844 | 69948866 | - |
| intron:TMEM245 | ACTTCATCTAACGAGATTAAAAG | ACTACATTTAACTAGATTAATAA | ...\*...\*....\*....... | 3 | 1.034177215 | chr9 | 109081965 | 109081987 | + |
| intron:ACVR1C | ACTTCATCTAACGAGATTAAAAG | AATTCACCTAAAGAGATTAACAT | .\*....\*....\*........ | 3 | 0.945879111 | chr2 | 157578900 | 157578922 | - |
| intron:DIO2-AS1 | ACTTCATCTAACGAGATTAAAAG | TCTACATTTAATGAGATTAAGAT | \*..\*...\*...\*........ | 4 | 0.703915663 | chr14 | 80414546 | 80414568 | + |
| exon:PM20D2 | ACTTCATCTAACGAGATTAAAAG | CCTTCATTAAAAGAGATTAAAAG | \*......\*\*..\*........ | 4 | 0.43009247 | chr6 | 89162311 | 89162333 | - |
| exon:ACTL6A | ACTTCATCTAACGAGATTAAAAG | ACTTCATCTGAAAAGATTAAAGA | .........\*.\*\*....... | 3 | 0.406822905 | chr3 | 179569808 | 179569830 | - |
| exon:RNF217 | ACTTCATCTAACGAGATTAAAAG | ACCTCATTTAAGGAGATTAGTGC | ..\*....\*...\*.......\* | 4 | 0.320295198 | chr6 | 125076821 | 125076843 | + |
| exon:UBN2 | ACTTCATCTAACGAGATTAAAAG | AATTCATCATACTAGATTAAAAA | .\*......\*\*..\*....... | 4 | 0.311578392 | chr7 | 139258476 | 139258498 | - |
| exon:ACOT13/C6orf62 | ACTTCATCTAACGAGATTAAAAG | ATTTCATATAACTAGAGTAAAAT | .\*.....\*....\*...\*... | 4 | 0.23590875 | chr6 | 24704115 | 24704137 | - |
| exon:FLVCR1-AS1 | ACTTCATCTAACGAGATTAAAAG | ACTACATCTTAAGAGATTCAGAA | ...\*.....\*.\*......\*. | 4 | 0.225999585 | chr1 | 212852139 | 212852161 | + |
3

## Slide 4
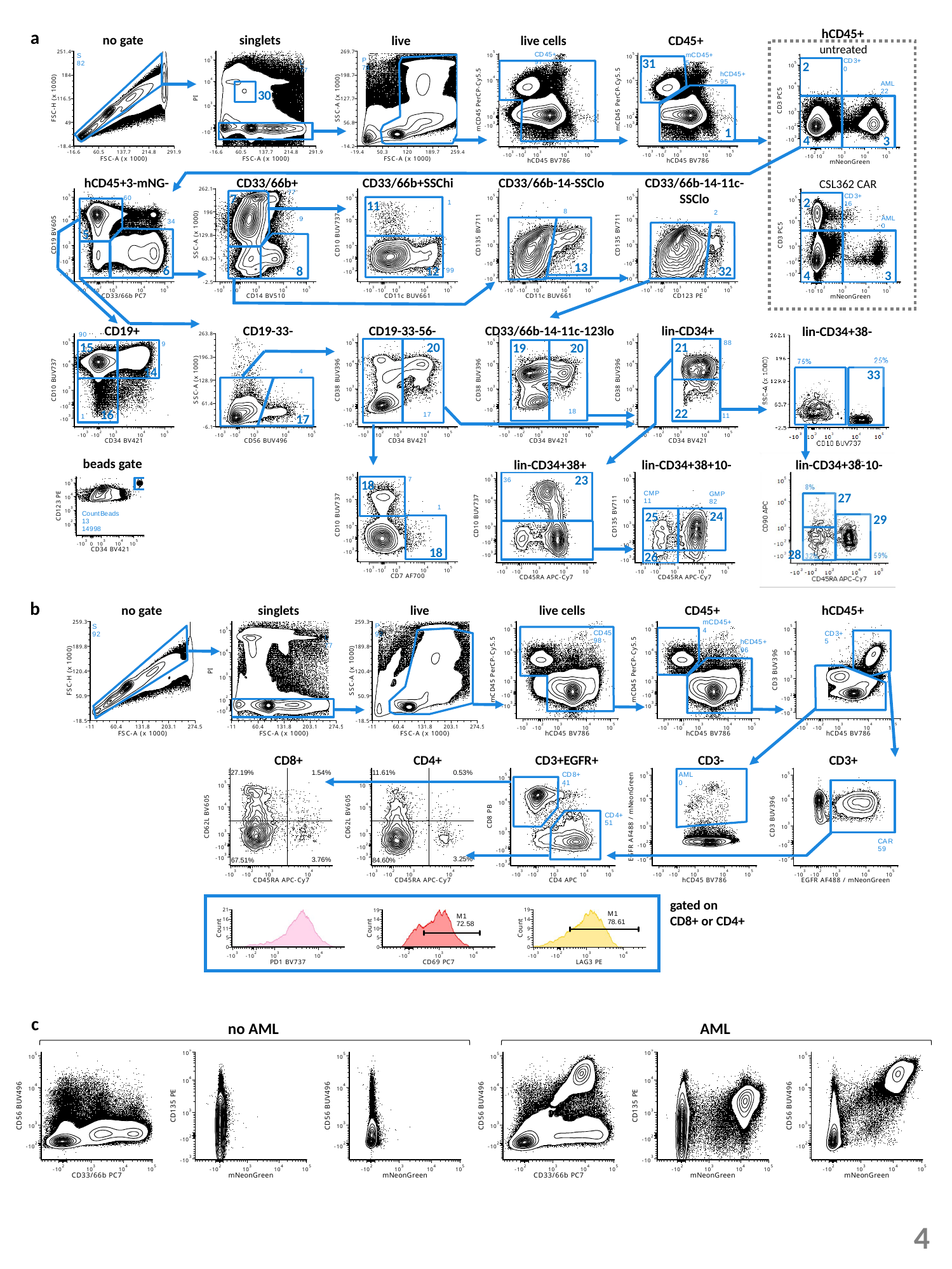

hCD45+
no gate
singlets
CD45+
live cells
live
untreated
31
2
30
1
4
3
hCD45+3-mNG-
CD33/66b+
CD33/66b+SSChi
CD33/66b-14-SSClo
CD33/66b-14-11c-SSClo
CSL362 CAR
7
2
11
5
13
6
12
8
32
3
4
CD19+
CD19-33-
CD19-33-56-
CD33/66b-14-11c-123lo
lin-CD34+
lin-CD34+38-
21
15
20
19
20
14
33
22
16
17
beads gate
lin-CD34+38+
lin-CD34+38+10-
lin-CD34+38-10-
23
18
27
24
25
29
18
28
26
a
b
no gate
singlets
live
live cells
CD45+
hCD45+
CD8+
CD4+
CD3+EGFR+
CD3-
CD3+
gated on
CD8+ or CD4+
c
no AML
AML
4
